# Positivity rates, trends and experiences of health workers on human papillomavirus screened using genexpert in Uganda: a three-year retrospective cohort study

**DOI:** 10.1186/s12885-024-12141-9

**Published:** 2024-03-25

**Authors:** Harriet Nakigozi, Paul Mukama Ategyeka, Susan Nabadda, Rawlance Ndejjo

**Affiliations:** 1 National Health Laboratory and Diagnostic Services, Ministry of Health, Kampala, Uganda; 2 School of Public Health, College of Health Sciences, Makerere University, Kampala, Uganda

**Keywords:** Human papilloma virus (HPV), Cervical cancer, Screening, Genexpert, Genotype

## Abstract

**Introduction:**

Cervical cancer is a major public health issue in Uganda, with high incidence due to limited screening especially in rural areas. In 2019, HPV DNA testing using GeneXpert was rolled out to improve screening access. Assessing progress and challenges since its introduction is important.

**Aim:**

To determine genotype distribution and explore health worker experiences with HPV screening using GeneXpert in Uganda.

**Methods:**

We conducted a retrospective cohort study where HPV screening data from 66 GeneXpert labs from March 2021-May 2023 country wide was analyzed. We used descriptive statistics to provide percentages and proportions from the data. Seven focus group discussions and five interviews were done with health workers to understand experiences.

**Results:**

We extracted 24,497 HPV tests that were done, and 39.1% were HPV positive. Other high-risk HPV genotypes were the most common at 65%, followed by HPV 16 (17%) and HPV 18/45 (18%). 15% of the HPV positive cases had more than one genotype. Qualitative findings showed inconsistent health worker knowledge, high workload, and complex care seeking behaviors as main challenges. It also revealed low community awareness, care seeking from traditional healers,

**Conclusion:**

HPV DNA testing has been expanding since its rollout, but the yield of HPV cases is lower than expected, signaling need to address supply-side challenges. Limited information on HPV among health workers especially community health workers, demand-side barriers like myths, medical pluralism and social norms must also be tackled through trainings of health workers and awareness campaigns engaging communities. Although access to GeneXpert services has increased, health system weaknesses pose bottlenecks to screening HPV. Targeted interventions are required to strengthen HPV diagnosis, prevent cervical cancer and save lives.

## Introduction/ background

Globally, an estimated 604,000 cases of cervical cancer were diagnosed and 340,000 women died from it in 2020 [1, 2]. Cervical cancer ranks as the fourth most frequently diagnosed leading cause of cancer death among women worldwide and in Africa, cervical cancer contributes to approximately a quarter of those deaths [3]. High risk human papillomavirus is the causative agent of cervical cancer and it is mostly common in Sub– Saharan Africa [4].

Cervical cancer screening programs have been successful in reducing the cervical cancer burden in most high-income countries [5].However many of the countries in sub-Saharan Africa (SSA) have not been able to establish and sustain screening programs due to financial, logistical and socio-cultural barriers, among other challenges [1]. The incidence of cervical cancer is 54.8 per 100, 000 and death is 40.5 per 100,000 in Uganda [6]. The high incidence can be explained by the challenges that limit cervical cancer screening among women especially in rural areas. To improve cervical cancer screening in Uganda, the government rolled out screening of HPV by genexpert in 2019.

The WHO has prioritized HPV testing over simpler visual inspection with acetic acid (VIA) for secondary prevention, where resources permit, due to higher test accuracy, longer screening interval and its compatibility with self-collection [7]. This has also led to scale-up of HPV screening where it is now possible to diagnose HPV by genotypes on existing large footprints of nucleic acid amplification test (NAAT) platforms which already exit in many LMICs Uganda inclusive.

In 2019, a pilot study was implemented to assess the feasibility of HPV testing services across five sub-Saharan African countries with Uganda included [7]. The goal of the study was to describe the service delivery approaches that enable access to integrated HPV testing using existing NAAT platforms. Therefore, this study assessed the progress of HPV testing services using genexpert by determining the prevalence, geno-type distribution and barriers/ challenges faced by health workers in HPV diagnosis using genexpert.

## Methods

### Study design

The study was a retrospective cohort study that employed both quantitative and qualitative research methods. Qualitative data (key informant interviews (KIIs) and focus group discussions (FGDs)) was collected among community health workers, nursing officers and laboratory personnel. The health workers selected to participate in the study provided HPV screening, and diagnostic services for women using the GeneXpert machines. We used the qualitative methods for triangulation purposes with the secondary data. The quantitative methods involved reviewing records of women that were screened for HPV across the country from March 2021 to May 2023. The data was extracted from the LabXpert that generates data from the GeneXpert machines.

### Study setting

The study was conducted in all the 66 diagnostic centres/genexpert sites diagnosing HPV across the country. HPV data was extracted from the LabXpert and then analyzed. Furthermore, seven focus group discussions and five key informant interviews were conducted.

### Study Population

The study population comprised of all women who screened for HPV using the GeneXpert machine across the country at GeneXpert sites during the period of March 2021 to May 2023. These patients included those who were documented in labxpert as having screened for HPV. This period was chosen because screening of HPV using the GeneXpert machine was started in 2021 with only 10 genexpert sites that diagnosed HPV and later that year it was expanded to other regions in the country.

### HPV DNA testing using GeneXpert assay

Genexpert is a DNA molecular testing platform using fluorescence to detect the presence of the high risk or (oncogenic) types of HPV. The machine reports only three types of HPV this include HPV 16, HPV 18/45 and other high-risk HPV (hrHPV) in a single run. Cervical specimens in Thin Prep™ Pap test vials containing PreservCyt™ Solution were used for testing with the Genexpert HPV 16, 18/45 and other hrHPV assay. A volume of 1 m was used and was pipetted and put into the HPV genexpert cartridge then after the cartridge was inserted in the loading sample bay of the machine and the test was started automatically. Results were released after 1 h.

Genexpert HPV assay detects hrHPV infections of the following types: HPV 16, HPV 18/45; and reports 11 other high-risk types in pooled results in less than one hour. The Xpert HPV Assay is a qualitative in vitro test for the detection of the E6/E7 region of the viral DNA genome from 14 high risk HPV types in a single analysis. Genexpert HPV assay specifically identifies types HPV16 and HPV 18/45 in two distinct detection channels, and reports 11 other High-risk types (31, 33, 35, 39, 51, 52, 56, 58, 59, 66 and 68) in a pooled result [8]. It also reports Errors and Invalid results.

The machine can also report Invalid as a result: this indicates a problem with the sample or PCR reaction itself therefore, the test cannot be completed and no result is available. Common causes of errors include insufficient sample volume, PCR inhibition, or issues with reagents/cartridge. Thus, Invalid means no result due to sample/assay issues.

The machine can also report Error as a result: this indicates a mechanical or software problem with the GeneXpert device. It shows that the test process was interrupted and no diagnostic result is obtained. Common errors include power failure, cartridge/module motion errors, or processing errors. Thus, Error means no result due to device-related issues.

An invalid result requires retesting with a new sample. An error code may require troubleshooting or restarting the device before retesting the same sample. Interpreting invalid vs. error results can help identify appropriate corrective actions.

### Data collection procedure

Data was downloaded into Microsoft Excel from the labxpert that generates data from the genexpert machines. The data was then exported to Stata v14 where data cleaning was done. The identified duplicates within the data were dropped from the data set. For the qualitative component, women were selected to participate in the seven focus group discussions while five health workers who worked at the central public laboratory on HPV diagnosis were selected for key informant interviews. The health workers were asked questions on the challenges they face during diagnosis and how they overcome some of these challenges. These interviews were audio recorded and key notes taken during the interviews.

### Data analysis

Data was analyzed using Stata v.14 software and excel where descriptive statistics was done by providing frequencies and proportions. Then the data was presented using tables and graphs. Trends of HPV cases diagnosed using genexpert machines over the three years were shown using line graphs. Thematic analysis was done with the help of Atlas ti V22. Themes were presented with their respective quotations.

## Results

**Fig. 1:**
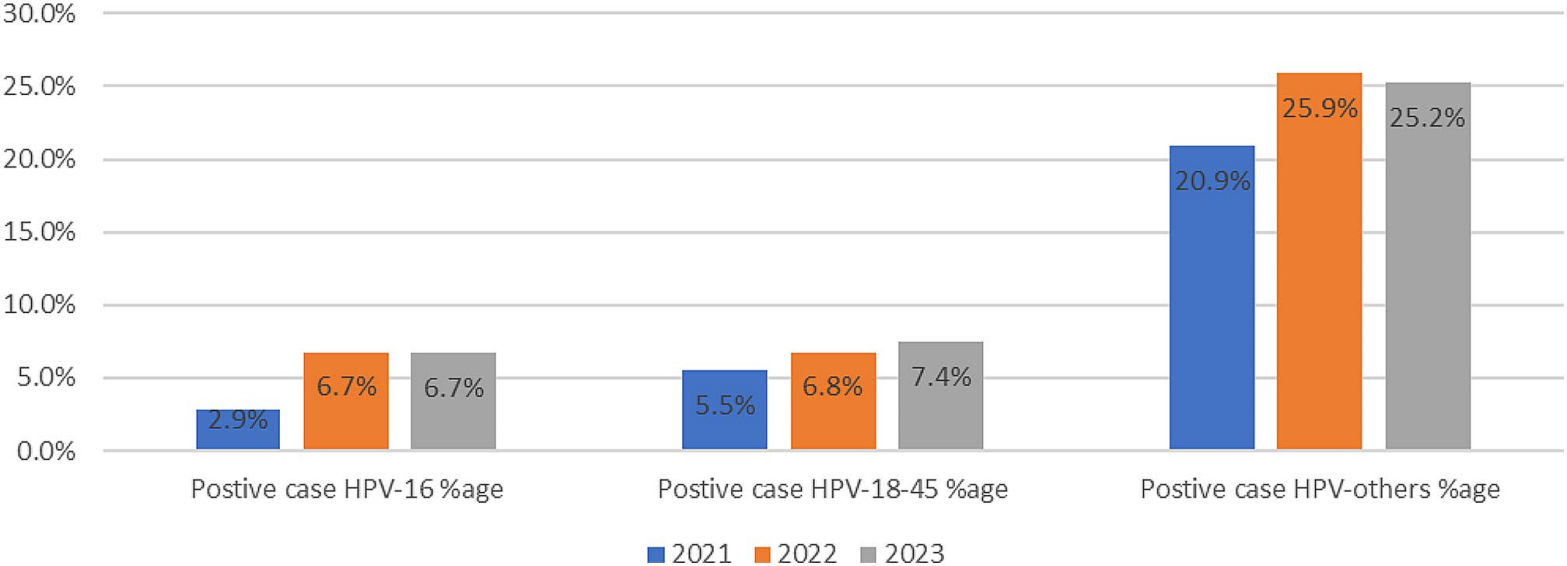
Shows HPV results by genotype over 3 years

A total of 24,497 tests were done with GeneXpert machines to diagnose HPV for a period of three years that is 2021, 2022 and 2023. Positive cases diagnosed were 39.1% (9590/24,497), at (95%CI 37.6 − 40.7%) errors during diagnosis were 4.0% (978/24,497) at (95%CI 3.8 − 4.2%), invalid results were 3.5% (857/24,497) at (95%CI 3.3 − 3.7%), and no results were 0.63% (154/24,497) at (95%CI 0.44 − 0.82%), as seen in Table 1; Fig. 1.

**Fig. 2:**
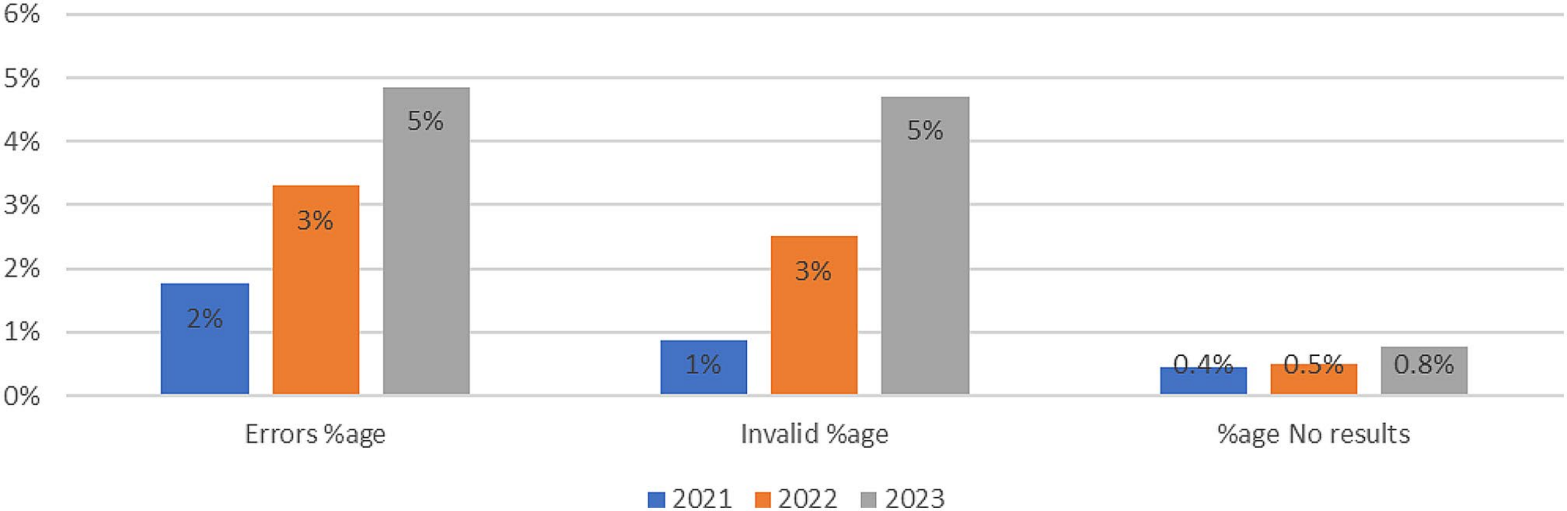
Invalid, errors and no results for HPV using the genexpert machine over the 3 years

In Table 2, out of 24,497 tests done (39.1%) 9,590 positive HPV cases were diagnosed, where 17% (1624/9590) (95%CI 15.7 − 18.2%), were HPV-16, 18% (1726/9590) (95%CI 16.7 − 19.2%), were HPV 18–45 and 65% (6240/9590) at (95%CI 63.0 − 67.0%), were other HPV genotypes as seen in Table 2. Only 15.04% (1,442/9590) at (95%CI 13.9 − 16.1%), of the HPV cases had multiple genotypes. Figure 2: In 2022, more positive cases were diagnosed because more tests were done compared to 2021 and 2023 with other genotypes being the most diagnosed during all the three years.

**Table 1:** Showing distribution of HPV tests done by GeneXpert

Year	Neg	Pos	Error	Invalid	No results	Total
2021	308	133	8	4	2	455
2022	6891	4984	418	318	63	12,674
2023	5719	4473	552	535	89	11,368
Total	12,918	9590	978	857	154	24,497

**Table 2:** Showing HPV positive cases by genotype distributed by year of diagnosis

HPV genotype	2021	2022	2023	Total
HPV − 16	13	851	760	1624
HPV- 18–45	25	856	845	1726
HPV -Others	95	3277	2868	6240
Total	133	4984	4473	9590

**Fig. 3:**
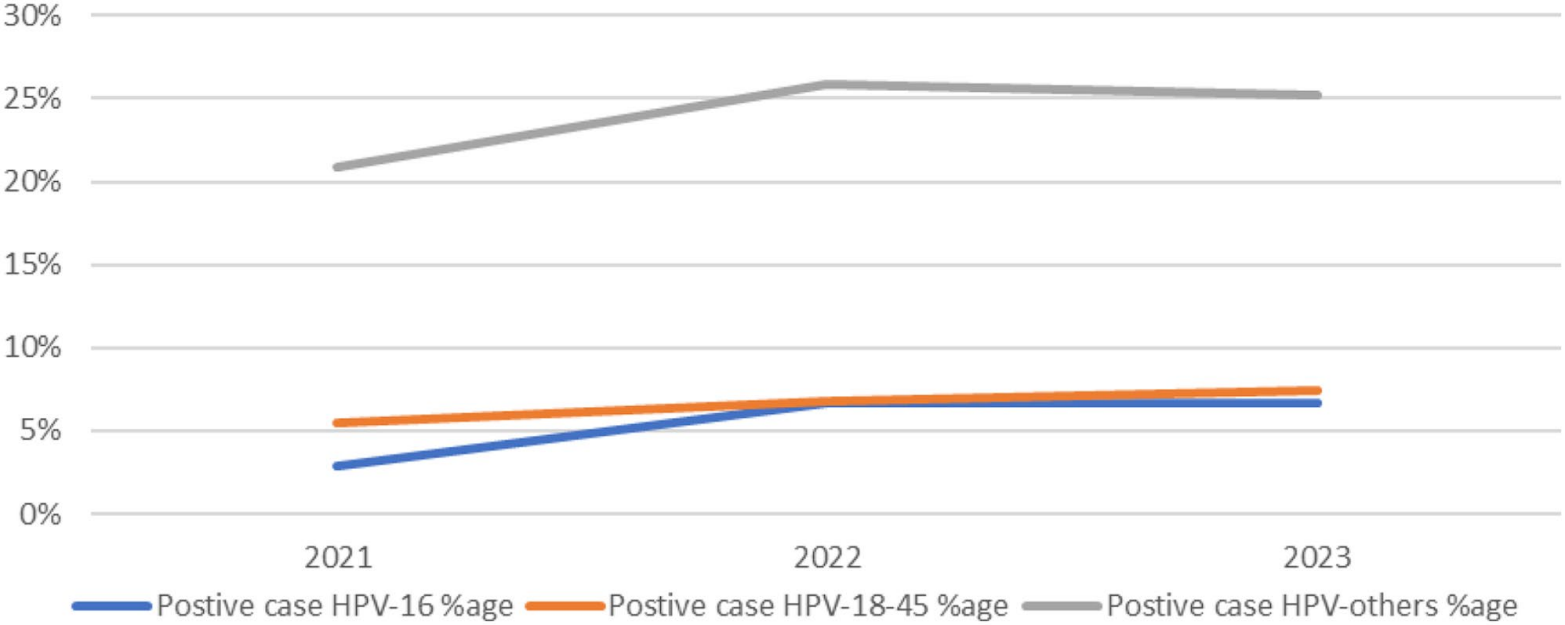
Trend of HPV positive cases over the three years

Figure 3 shows a slightly upward trend of the three genotypes starting 2021 (when the screening tool was introduced) to 2023, that were positively diagnosed using genexpert.

### Experiences of health workers on screening and diagnosing HPV

#### Key findings

Generally, there was low awareness of cervical cancer infections or HPV infections among people and community health workers. There are lots of misconceptions on cervical cancer related infections in spite of the perceived knowledge. Therefore, few women will turn up for screening services. This in turn reduces the chances of prevention since HPV screening is a prevention method for cervical cancer. Which may lead to under-utilization of the genexpert machine thus expires of consumables or supplies such as cartridges.

*“Even in trainings we get as community health workers, we have not been taught anything concerning diseases of the cervix or cervical cancer”. (FGD 1, NH (CHW))*

Informal care seeking. When women get infected with HPV or have health issues, they will seek care in informal settings such as traditional healers and as they do, their condition worsens and that is when they are sent to the hospitals. Yet they would have prevented the disease if they sought screening services earlier.

*“unless you seek traditional medication, you cannot go to hospital and get healed from cancer” (FGD 3 NM)*.

*“You go to a health facility or centre where they prescribe for you a medication which you cannot afford to buy. Instead, you choose to put herbs hoping that by luck you will recover’’ (FGD 2, NR).*

Most of the health workers were knowledgeable of the HPV symptoms: Nurses could identify manifestations of HPV or cervical cancer disease but could not relate their theoretical knowledge to practice. Though, there was lack of uniform understanding of cervical cancer guidelines even among those who had trained.

*“In the last training of cervical cancer, we were told that insertion of speculum or visual inspection with acetic acid does not matter or not helpful. On contrary it can be the cause of complications or pain when treating mothers” (FGD HCW 2)*.

Most of the health workers found that there was a high work load due to the wide variation in frequency of cases seen and not all sick or HIV positive patients seek care at health facilities.

*“Till now, I haven’t encountered a case of cervical cancer but patients with cervical cancer are many in the health centre. Cervical cancer is infection of cervix with foul smelling discharge” (FGD1 KGH)*.

Care seeking practices are still lacking or inadequate due to challenges such as medical pluralism among people in various communities or regions of Uganda. Medical pluralism was found to be a common practice. The communication network determines choice of care seeking.

*“some people first seek advice from older people who then tell them what to do either to go to hospital or for traditional treatment” (FGD4 KMG).*

## Discussion

This study shows the progress of HPV screening using the genexpert machine since it’s scaling up after the pilot study that was conducted for 7 months at 10 genexpert sites in 2019. Using data from the Labxpert it shows that 39.1% of the women screened for HPV were positive. This number is relatively lower compared to a study conducted in Greece that showed 43.9% of the women that were screened for HPV were positive [9]. For the pilot study conducted in sub Saharan Africa, the prevalence of positive HPV cases screened using genexpert was much lower at 28% [7]. The prevalence of HPV among men was also lower according to WHO compared to the finding in this study; it showed a global hrHPV pooled prevalence of 21% in 2023 [10].

This low prevalence can be explained by the challenges which can be faced especially when a new diagnostic or screening tool is introduced these challenges may include limited lab supplies, limited trained personnel.

HPV other genotypes was the most prevalent at 65% compared to HPV 16 and HPV 18/45. These results are similar to the results from the pilot study [7] that showed that HPV other genotypes was the most prevalent where the prevalence ranged from 44 to 83% in the sub-Saharan countries (Malawi (77%), Nigeria (83%), Senegal (44%), and in Uganda (76%)). Though these findings are contrary to the findings of a study conducted in Greece that showed HPV 16 was the most high-risk genotype compared to others [11].

In this study, 15% of the women who screened positive for HPV had multiple HPV genotype infections with most suffering from other HPV genotypes and HPV 16. A study in China showed that the prevalence of multiple HPV genotype infection was at 19.3% which is slightly higher than the 15% found in this study [12].

The prevalence of errors and invalid results were 4%and 3.5% and these may have occurred due the quality of the sample or power outages in the country. These findings are similar to findings from Sub-Saharan countries and low– resource settings that showed the prevalence ranging from 0 to 8% [7, 13].

The study revealed that health workers especially the community health workers have limited knowledge about screening and diagnosing HPV in the communities. A major challenge was low awareness and misconceptions about HPV and cervical cancer among both community members and some health workers. False beliefs such as contracting cervical cancer from poor hygiene were common. This lack of knowledge likely contributes to low screening uptake. This is similar to a study conducted in Tanzania that showed the need for training community health workers in HPV screening and care [14].

Another key finding was the preference for informal care such as traditional healing, even for conditions like cervical cancer. Factors driving medical pluralism and care seeking from traditional practitioners first include cultural beliefs, high cost of care at health facilities, long waiting times, and perceived poor quality of care. Strategies to improve cervical cancer awareness must engage traditional healers and community stakeholders to address misconceptions and build trust in the health system. A study conducted in Ghana showed that most women preferred to visit traditional healers which is similar to these study findings [15].

The study also highlighted inconsistencies in health workers’ knowledge about cervical cancer guidelines, with some providers demonstrating inaccurate information and practices. This knowledge gap limits their ability to appropriately screen and diagnose HPV. Regular training and mentorship are needed to strengthen providers’ competencies in this area. A study conducted in Eswatini showed similar results siting that with a deficit of knowledge among health workers on HPV screening and care can result in inaccurate information being communicated to clients [16].

Moreover, the variation in frequency of seeing cervical cancer cases makes it difficult for providers to gain and maintain expertise. But the burden of cervical cancer regionally remains high. Thus, health systems factors like staffing and resources for screening must be strengthened. Similar challenges of increased work load among health workers on HPV screening and care were faced in Tanzania [17].

Finally, complex care seeking behaviors, including advice from social contacts, underscores the need for comprehensive cervical cancer communication at the individual, family, and community levels. Mass media campaigns and community outreach by health workers can help dispel myths and shape positive health practices around cervical cancer prevention. These findings are similar to those found in a study conducted in South Africa where the decision for HPV screening and seeking care can be influenced by the social cycles of the woman that is to say women are easily influenced by the peers that can encourage them to screen for HPV [18].

## Conclusion

With increased support with resources such as trained personnel, laboratory supplies and increased health education on prevention of HPV, HPV screening services can be easily accessed even in rural areas of Uganda because of the availability of genexpert machines that can easily be used to screen for HPV. The study also provides valuable insights into the experiences by health workers on HPV and cervical cancer control from both the demand and supply sides. Addressing awareness, capacity building, health systems weaknesses and harmful norms is key to increasing HPV screening services.

## Data Availability

Data are available upon request from the corresponding author. Data is available on a national database called LabXpert - ntrl.or.ug/labxpert or https://labxpertds.com/ and can be accessed from ministry of health upon request.
